# Cumulative antibiogram at a tertiary cancer center in Jordan showing alarming antimicrobial resistance trends

**DOI:** 10.1017/ash.2026.10888

**Published:** 2026-10-09

**Authors:** Sawsan Mubarak, Iyad Sultan, Osama Abu Ata, Omar Khresat, Hadeel AlGhawrie

**Affiliations:** King Hussein Cancer Center, Amman, Jordan

## Abstract

**Objective:**

Multidrug-resistant (MDR) bacteria poses a significant threat to patient care, especially in vulnerable populations. This study evaluates antimicrobial resistance (AMR) trends at a tertiary cancer center in Jordan, analyzing data from 2007 to 2022.

**Design and settings:**

This retrospective study analyzed 51,697 first bacterial isolates from 108,432 cultures at King Hussein Cancer Center (2007–2022). Data were stratified into three periods and processed using the AMR package for R. Binomial logistic regression models were employed to forecast resistance trends to 2030.

**Results:**

The comprehensive analysis of 51,697 first bacterial isolates revealed that 61% were Gram-negative bacteria (GNB) and 39% were Gram-positive bacteria (GPB), with *Escherichia coli*, coagulase-negative *staphylococci* (CoNS), and *Klebsiella pneumonia* predominating. MDR was identified in 36.5% of isolates, with GNB accounting for 84.4%, while extensively drug-resistant (XDR) was confined to *Acinetobacter baumannii*-calcoaceticus and *Pseudomonas aeruginosa*. Conversely, nonfermenting GNB showed declining resistance trajectories for piperacillin/tazobactam and amikacin. Among Gram-positives, methicillin-resistant *Staphylococcus aureus* (MRSA) surged from 37% to 57%. Vancomycin-resistant *enterococci* (VRE) increased reaching 13%. Blood cultures were predominantly GPB (65%), while respiratory and urine samples were primary sources of resistant GNB. Hematologic malignancy patients exhibited the highest extensively drug-resistant (XDR) rates (5.6%–7.9%) and VRE burden (32% in leukemia). Predictive models forecast escalations by 2030: MRSA 72.7%, VRE in *Enterococcus faecium* 70%, and cefepime resistance in *Enterobacterales* 61.9%.

**Conclusion:**

Our AMR patterns are alarming and vary significantly by specimen site and cancer diagnosis, underscoring the urgent need for enhanced stewardship, infection control, and diagnosis-specific empirical protocols.

## Introduction

Antimicrobial resistance (AMR) poses a significant global health threat, particularly within human medicine, where it imposes substantial burdens on patients and healthcare systems. The U.S. Centers for Disease Control and Prevention (CDC) and the World Health Organization (WHO) categorize AMR pathogens as a threat to human health, with more than 2 million AMR infections and 29,000 deaths occurring annually in the United States alone, at an attributable cost exceeding ＄4.7 billion.^1^

AMR poses a significant global health threat, imposing substantial burdens on patients and healthcare systems; the CDC and WHO both categorize AMR pathogens as a top threat to human health, with more than 2 million AMR infections and 29,000 deaths occurring annually in the United States alone, at an attributable cost exceeding ＄4.7 billion.^1^ Cumulative antibiograms, periodic, institution-level summaries of antimicrobial susceptibility compiled by microbiology laboratories in collaboration with infection control teams, remain essential tools for tracking resistance trends, guiding stewardship, and informing empiric antibiotic therapy, particularly for severely immunocompromised patients such as those who are neutropenic, have hematologic malignancies, or have undergone hematopoietic stem cell transplantation.^2–6^

In 2011, an international expert panel (the Clinical and Laboratory Standards Institute (CLSI), the European Committee on Antimicrobial Susceptibility Testing, and the United States Food and Drug Administration) established consensus definitions for multidrug-resistant (MDR: non-susceptible to ≥ 1 agent in ≥ 3 antimicrobial categories), extensively drug-resistant (XDR: non-susceptible to ≥ 1 agent in all but ≤ 2 categories), and pandrug-resistant (PDR: non-susceptible to all agents in all categories) bacteria.^7^ Notably, six bacterial species are associated with highest rates of bacterial resistance, namely MDR bacteria. These are the *Enterococcus faecium, Staphylococcus aureus, Klebsiella pneumoniae, Acinetobacter baumannii, Pseudomonas aeruginosa,* and *Enterobacter* species; collectively known as ESKAPE pathogens.^1^ Constructing a reliable antibiogram requires careful exclusion of duplicate and surveillance cultures, accurate susceptibility reporting aligned with current breakpoints, and well-trained microbiology staff; real-time, EMR-integrated antibiogram generation remains unavailable in many institutions, particularly in low- and middle-income countries.^8,9^

Our institution is the leading tertiary cancer center in Jordan, a 340-bed facility that prepares and distributes hospitalwide antibiograms annually to guide empirical treatment. This descriptive epidemiological surveillance study addresses 16 years (2007–2022) of cumulative antibiogram data from a tertiary cancer center in Jordan to characterize temporal trends in bacterial isolate distribution and the prevalence of MDR, XDR, and PDR phenotypes across three defined study periods; to describe resistance patterns stratified by pathogen group (*Enterobacterales*, non-fermenting Gram-negative bacilli, and Gram-positive organisms), specimen source, and cancer diagnosis; to model and forecast resistance trajectories for key pathogen-antimicrobial pairings through 2030 using binomial logistic regression; and to identify diagnosis- and site-specific patterns that can inform targeted empirical therapy, antimicrobial stewardship, and infection control strategies at our institution and in comparable resource-limited oncology settings.

## Methods

### Study design and setting

A descriptive analysis of cumulative antibiogram at King Hussein Cancer Center (KHCC) over a 16-year period (2007–2022), designed to characterize resistance trends and generate hypothesis-generating predictive forecasts rather than to test a prespecified causal hypothesis. The 16-year period was divided into three approximately equal ∼5-year intervals (2007–2012, 2013–2017, and 2018–2022) to enable balanced evaluation of temporal trends in bacterial prevalence and resistance.

### Data collection and processing

Following IRB approval, microbial culture files from 2007 to 2022 were retrieved from our infection control program’s secure repository. Each file contained patient medical record numbers, specimen site and type, collection date, admission date, age group (adult/child), and antimicrobial susceptibility results (resistant, susceptible, intermediate).

Records were linked to the cancer registry via medical record numbers, with specimen sources standardized into 10 categories and blood cultures classified as peripheral or central line-derived; surveillance swabs, stool cultures, and non-bacterial isolates were excluded. Following CLSI M39 guidelines, only diagnostic isolates from species with ≥30 isolates were included, using the first isolate per patient per year, with MDR/XDR/PDR phenotypes classified per Magiorakos et al.^3,7^ To account for CLSI breakpoint revisions over the 16-year period, raw MIC values were retrospectively re-interpreted against a single, current breakpoint standard, ensuring susceptibility classifications were internally consistent across all three study periods rather than reflecting breakpoint-driven reclassification; susceptibility testing was performed using automated systems (VITEK, bioMérieux) throughout most of the study period.

### Antibiogram construction

The cumulative antibiogram summarizes antimicrobial susceptibility testing results, reporting the percentage of isolates susceptible (%S) to each organism-antimicrobial pairing. Specific antimicrobial panels reported for each organism adhered to the National Healthcare Safety Network (NHSN) requirements, using the following formula.^10^

$$\%S=\frac{Number\;isolates\;tested}{Suceptible\;Number\;of\;isolates\;tested}\;*\;100\%\;$$

### Statistical analysis

The R-AMR package was utilized to standardize microorganism and antimicrobial agent nomenclature, using intelligent algorithms to code all microorganisms and antimicrobials according to standard taxonomies.^2^ For selected pathogen/ antimicrobial pairings of clinical interest, binomial logistic regression models were fit at the isolate level, with resistance status (resistant vs susceptible) as the binary outcome and calendar year (2007–2022) as the sole continuous predictor. Predicted resistance probabilities for 2030 were obtained by extrapolating each fitted model, with 95% confidence intervals derived from the standard error of the linear predictor on the logit scale and back-transformed to the probability scale; the reported *P* value reflects the significance of the year coefficient. All statistical analyses were conducted using R software (v.4.0.5), and visualization was performed using the ggplot2 package.^11^

## Results

### Bacterial isolates prevalence and temporal trend

Over the 16-year study period; 51,697 first bacterial isolates were analyzed, of which 61% (N = 31,781) were GNB and 39% (N = 19,916) were GPB. The most common bacterial isolates overall were *E. coli* (13,159; 25%), Coagulase-negative staphylococci (CoNS) (8,702; 17%), *Klebsiella pneumoniae* (5,075; 10%), *Pseudomonas aeruginosa* (3,673; 7.1%), *Staphylococcus aureus* (2,863; 5.6%), *Acinetobacter baumannii-calcoaceticus* (1,920; 3.7%), *Enterococcus faecalis* (1,517; 2.9%), viridans group streptococci (VGS) (1,265; 2.4%), and *Proteus mirabilis* (1,099; 2.1%). Dividing the study into three periods (2007–2012, 2013–2017, and 2018–2022) reveals temporal trends in bacterial prevalence, total isolates increased over time, driven by rising *E. coli* and *K. pneumoniae* while *A. baumannii-calcoaceticus* declined (Table 1).

**Table 1.** Most common bacterial isolates by study period (N (%) of total isolates per period) Table 1 long description.

Bacterial isolates	2007 to 2012	2013 to 2017	2018 to 2022
	N. (%)	N. (%)	N. (%)
*Escherichia coli*	3341 (33)	4125 (34)	5693 (33.5)
*Klebsiella pneumoniae*	1163(11.5)	1567(12.9)	2345 (13.8)
*Pseudomonas aeruginosa*	1048 (10.3)	1066 (8.8)	1559 (9.2)
*Staphylococcus aureus*	762 (7.5)	837 (6.9)	1264 (7.4)
*Acinetobacter baumannii-calcoaceticus*	706 (7.0)	529 (4.4)	685 (4.0)
*Proteus mirabilis*	240 (2.4)	362 (3.0)	497 (2.9)
*Enterococcus faecalis*	372 (3.7)	382 (3.1)	763 (4.5)
Viridans Group streptococci (VGS)	314(3.1)	397(3.3)	554(3.3)
Coagulase-negative *Staphylococci* (CONS)	2191 (21.6)	2876 (23.7)	3635 (21.4)

### Antimicrobial resistance trends

Over the 16-year study period, MDR bacteria accounted for 36.5% (N = 18,867) of all isolates, with GNB comprising 84.4% (N = 15,916) and GPB 15.6% (N = 2,951). The percentage of GPB (of total isolates) (38%, 39%, 40%), while GNB (62%, 61%, and 60%) showed slight fluctuations over the 3 study periods, as illustrate in Table 2. There was an increase in the percentage of MDRs in GNB moving from the first to the second period (34%, 39%, 39%), most species showed an increase in the percentage of MDR over time. XDR isolates constituted 5.4% (N = 1,733) of all GNB, limited to *A. baumannii-calcoaceticus* (N = 1,313) and *P. aeruginosa* (N = 420). No PDR isolates were identified throughout the study period.

**Table 2.** Antibiotic Resistance profiles across study periods Table 2 long description.

	2007–2012	2013–2017	2018–2022
Total Isolates	13294	16021	22382
Gram-Negative	8288	9869	13624
Percentage of total	62.3%	61.6%	60.9%
MDR	3,402 (41%)	3,576 (36.2%)	4,936 (36.2%)
XDR	678 (8.1%)	478 (4.8%)	577 (4.2%)
Gram-Positive	5006	6152	8758
Percentage of total	37.7%	38.4%	39.1%
MDR	568 (11.3%)	909 (14.7%)	1,474 (16.8%)

### Antimicrobial resistance patterns by pathogen group

**Figure 1.**
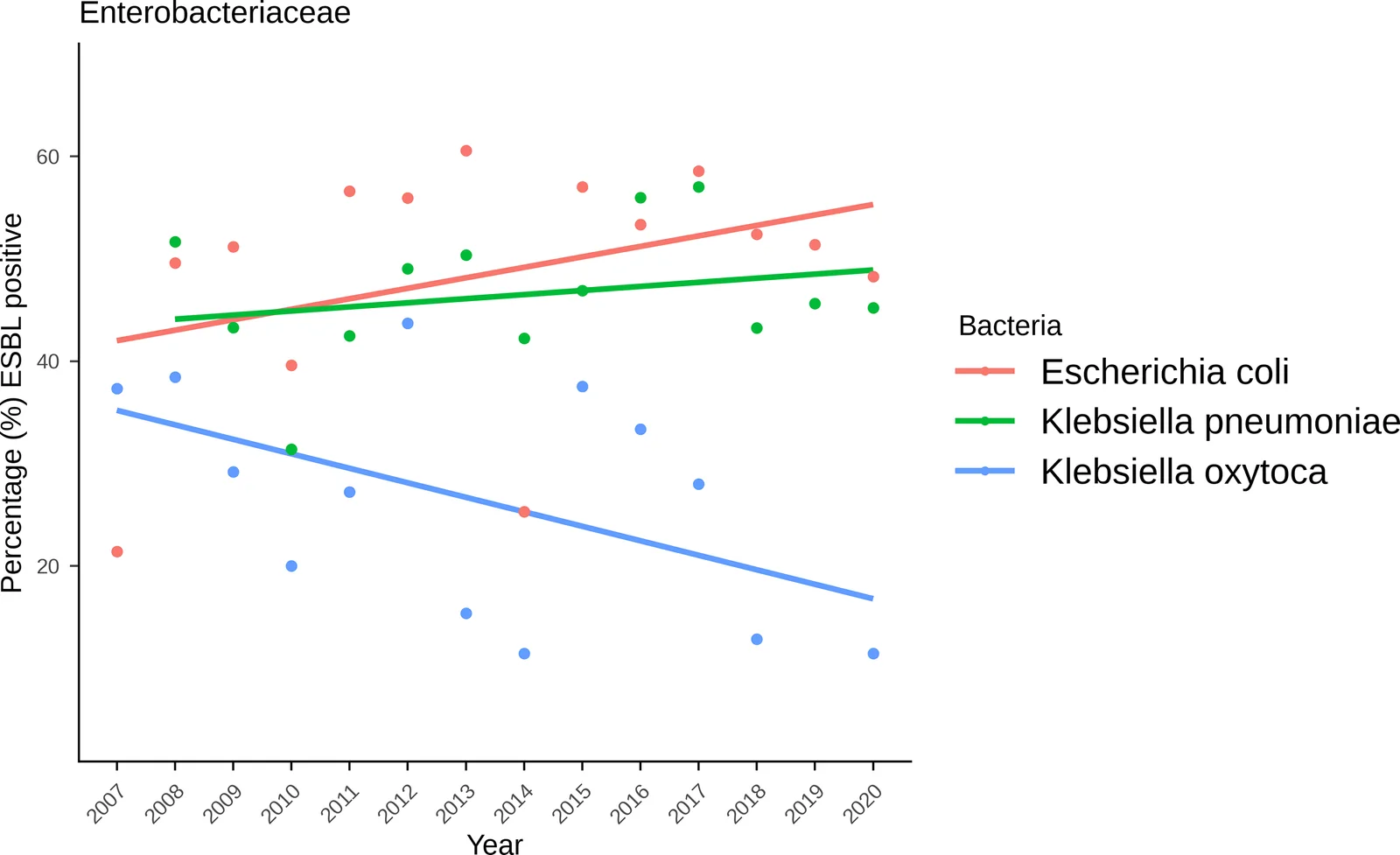
ESBL positivity trend among Enterobacteriaceae (2007–2020).

Among GNB, *Enterobacterales* were the most prevalent, comprising 20,928 first isolates. ESBL was detected in 45% of all *Enterobacterales*, predominantly in *E. coli* (47%), *K. pneumoniae* (43%), and *K. oxytoca* (2.5%), collectively accounting for 66% of MDR isolates among these three species, with ESBL positivity increasing over time in *E. coli* and *K. pneumoniae* (Figure 1). Cefepime resistance rose markedly (17%, 30%, and 37%) and carbapenem resistance showed the most concerning increase (0.2%, 1.8%, and 3.4%), while fluoroquinolone resistance remained persistently high (∼53%), aztreonam stable at 47%, and aminoglycoside resistance stable at 4%. No colistin resistance was detected in this family (Table 3).

**Table 3.** Key Antimicrobial Resistance Percentages by Study Period (Tested Isolates) Table 3 long description.

	2007–2012	2013–2017	2018–2022
Staphylococcus aureus			
Oxacillin resistance	37% (N = 730)	50% (N = 849)	57% (N = 1295)
Enterococcus species			
Vancomycin resistance	5% (N = 552)	13% (N = 615)	13% (N = 1094)
Enterobacteriaceae			
Amikacin	3% (N = 5278)	3% (N = 6455)	5% (N = 9150)
Cefepime	14% (N = 5215)	31% (N = 6401)	33% (N = 9049)
Ciprofloxacin	45% (N = 5218)	50% (N = 6396)	53% (N = 8986)
Piperacillin/tazobactam	7% (N = 5193)	19% (N = 6386)	12% (N = 9015)
Imipenem	0% (N = 5194)	2% (N = 6341)	3% (N = 8999)
NFGNB^*^			
Amikacin	40% (N = 1965)	37% (N = 1927)	35% (N = 2627)
Cefepime	42% (N = 1833)	33% (N = 1703)	31% (N = 2364)
Ciprofloxacin	45% (N = 1839)	36% (N = 1704)	35% (N = 2361)
Piperacillin/tazobactam	44% (N = 1964)	40% (N = 1927)	36% (N = 2627)
Imipenem	46% (N = 1941)	44% (N = 1904)	40% (N = 2609)
Colistin	1% (N = 1808)	1% (N = 1688)	1% (N = 1358)
Aztreonam	49% (N = 1713)	34% (N = 1291)	29% (N = 1823)

* NFGNB, Non-Fermenting Gram-Negative Bacilli.

Among 6,945 NFGNB isolates — predominantly *P. aeruginosa* (53%), *A. baumannii-calcoaceticus* complex (28%), and *S. maltophilia* (8.5%) —a favorable declining resistance trend was observed across aztreonam (53%, 37%, and 33%), cefepime (43%, 36%, and 30%), piperacillin/tazobactam (46%, 42%, and 35%), fluoroquinolones (57%, 48%, and 36%), aminoglycosides, and carbapenems (Table 3). Colistin resistance was documented in *A. baumannii-calcoaceticus* complex and *P. aeruginosa*, and all *B. cepacia* isolates (N = 36) were colistin resistant.

Among GPB, *S. aureus* oxacillin resistance rose to 57% in the last period, while CoNS did not meet the MDR definition. Of 2,248 *Enterococcus* spp. isolates, *E. faecalis* (67.4%) and *E. faecium* (29%) were most prevalent, with overall MDR rates of 61% — varying markedly between *E. faecalis* (49%) and *E. faecium* (90%). VRE prevalence increased from 5% to 13% and remained stable thereafter (Table 3).

### Antibiogram

A detailed antibiogram for all three study periods is provided in the Supplementary Table. The longitudinal analysis of the ten most prevalent isolates revealed significant resistance shifts over time. *A. baumannii-calcoaceticus* maintained a persistently high-resistance phenotype with critically low carbapenem susceptibility (19%–26%), though colistin retained near-universal activity (99–100%). Among *Enterobacterales*, cephalosporin efficacy declined notably, with *E. coli* ceftriaxone susceptibility dropping from 56% to 46% and *K. pneumoniae* cefepime susceptibility from 89% to 68%; carbapenem susceptibility remained robust for *E. coli* (≥92%) but showed a gradual downward trend for *K. pneumoniae*. In contrast, *P. aeruginosa* and *P. mirabilis* exhibited relatively stable profiles, with *P. aeruginosa* maintaining consistent susceptibility to piperacillin/tazobactam (84–88%) and amikacin (84–89%).

Among GPB, *S. aureus* oxacillin susceptibility declined markedly from 63% to 43%, reflecting rising MRSA prevalence, while clindamycin susceptibility remained stable (∼70%). CoNS exhibited consistently low oxacillin susceptibility (19–25%). Within *Enterococcus* spp., vancomycin susceptibility decreased from 95% to 87%. Despite these trends, last-resort agents — linezolid and teicoplanin — maintained near-universal activity (≈100%) across all GPB, including VGS, which remained fully susceptible to ampicillin and ceftriaxone throughout the study period.

### Antibiogram per site

Pathogen distribution varied significantly by specimen source: urine and respiratory cultures were predominantly GNB (84% and 81%, respectively), while blood isolates were predominantly GPB (65%). Resistance patterns similarly varied by site; oxacillin-resistant *S. aureus* peaked in respiratory (60%) and wound (58%) samples, and VRE accounted for 26% of *Enterococcus* spp. in blood cultures. Urinary *Enterobacterales* showed higher ciprofloxacin resistance but lower resistance to amikacin, piperacillin/tazobactam, and carbapenems, while respiratory samples were the predominant source of resistant NFGNB (Table 4).

**Table 4.** Antibiotic Resistance Profiles across Different Sites (% of site total) Table 4 long description.

Diagnosis	Urine	Blood	Wound	Respiratory	Others
Gram-Negative	83.80%	35.00%	48.00%	80.60%	42.60%
MDR	63%	53%	46%	35%	49%
XDR	2.2%	5.1%	7.6%	16%	2.2%
Gram-Positive	16.20%	65.00%	52.00%	19.40%	57.40%
MDR	62%	94%	81%	46%	87%
S.aureus					
Oxacillin resistance	52%	55%	58%	60%	56%
Enterococcus species					
Vancomycin resistance	12%	26%	16%	NA	21%
Enterobacteriaceae					
Amikacin	3%	7%	5%	6%	24%
Ciprofloxacin	55%	53%	49%	42%	46%
piperacillin/tazobactam	10%	17%	15%	18%	15%
imipenem	2%	5%	4%	6%	4%
NFGNB^*^					
Amikacin	26%	35%	30%	58%	35%
piperacillin/tazobactam	27%	37%	33%	62%	34%
imipenem	32%	39%	35%	64%	39%
colistin	0%	2%	1%	0%	4%

* NFGNB, Non-Fermenting Gram-Negative Bacilli.

### Diagnosis-specific resistance profiles

Resistance patterns varied significantly by cancer type (Table 5). While GNB proportions were broadly similar across diagnoses (54–63%), hematologic malignancies and bone/soft tissue sarcomas drove the highest XDR rates (5.6–8.3%). Leukemia patients showed particularly concerning profiles, including elevated VRE (32%), *Enterobacterales* amikacin resistance (11%), and NFGNB imipenem resistance (57%), markedly higher than the 37% seen in carcinoma patients. This pattern of heightened resistance in hematologic versus solid tumor patients was consistent across aminoglycosides, fluoroquinolones, and carbapenems. Lymphoma patients exhibited 7.9% XDR rates, while leukemia patients showed the highest MRSA prevalence (62%), closely followed by CNS tumor patients (61%). These findings underscore the need for diagnosis specific stewardship protocols, particularly for neutropenic fever management.

**Table 5.** Resistance Profiles by most common Cancer Diagnosis (% of tested isolates) Table 5 long description.

Diagnosis	Carcinomas	CNS	Leukemia	Lymphomas	Bone and soft tissue
Total	13215	887	1930	1593	863
Gram-Negative	8370	512	1090	882	465
Percentage	63.3%	57.7%	56.5%	55.4%	53.9%
MDR	4,314 (56%)	243 (52%)	640 (64%)	441 (54%)	209 (50%)
XDR	312 (4.0%)	20 (4.3%)	56 (5.6%)	64 (7.9%)	35 (8.3%)
Gram-Positive	4845	375	840	711	398
Percentage	36.7%	42.3%	43.5%	44.6%	46.1%
MDR	897 (19%)	61 (16%)	105 (13%)	95 (13%)	65 (16%)
S.aureus					
Oxacillin resistance	56% (N = 761)	61% (N = 64)	62% (N = 86)	54% (N = 98)	56% (N = 75)
Enterococcus species					
Vancomycin resistance	13% (N = 729)	17% (N = 35)	32% (N = 69)	20% (N = 61)	13% (N = 38)
Enterobacteriaceae					
Amikacin	4% (N = 5772)	4% (N = 323)	11% (N = 707)	8% (N = 577)	4% (N = 255)
Ciprofloxacin	53% (N = 5700)	45% (N = 320)	63% (N = 682)	57% (N = 556)	44% (N = 254)
piperacillin/ tazobactam	11% (N = 5726)	11% (N = 318)	19% (N = 679)	17% (N = 558)	11% (N = 253)
imipenem	3% (N = 5718)	3% (N = 318)	6% (N = 676)	4% (N = 558)	3% (N = 253)
NFGNB					
Amikacin	32% (N = 1534)	28% (N = 116)	51% (N = 228)	44% (N = 192)	38% (N = 129)
piperacillin/ tazobactam	35% (N = 1534)	29% (N = 116)	44% (N = 228)	45% (N = 192)	41% (N = 129)
imipenem	37% (N = 1529)	36% (N = 113)	57% (N = 226)	49% (N = 190)	45% (N = 128)
colistin	1% (N = 767)	0% (N = 60)	2% (N = 132)	1% (N = 104)	1% (N = 72)

### Trends and prediction

**Figure 2.**
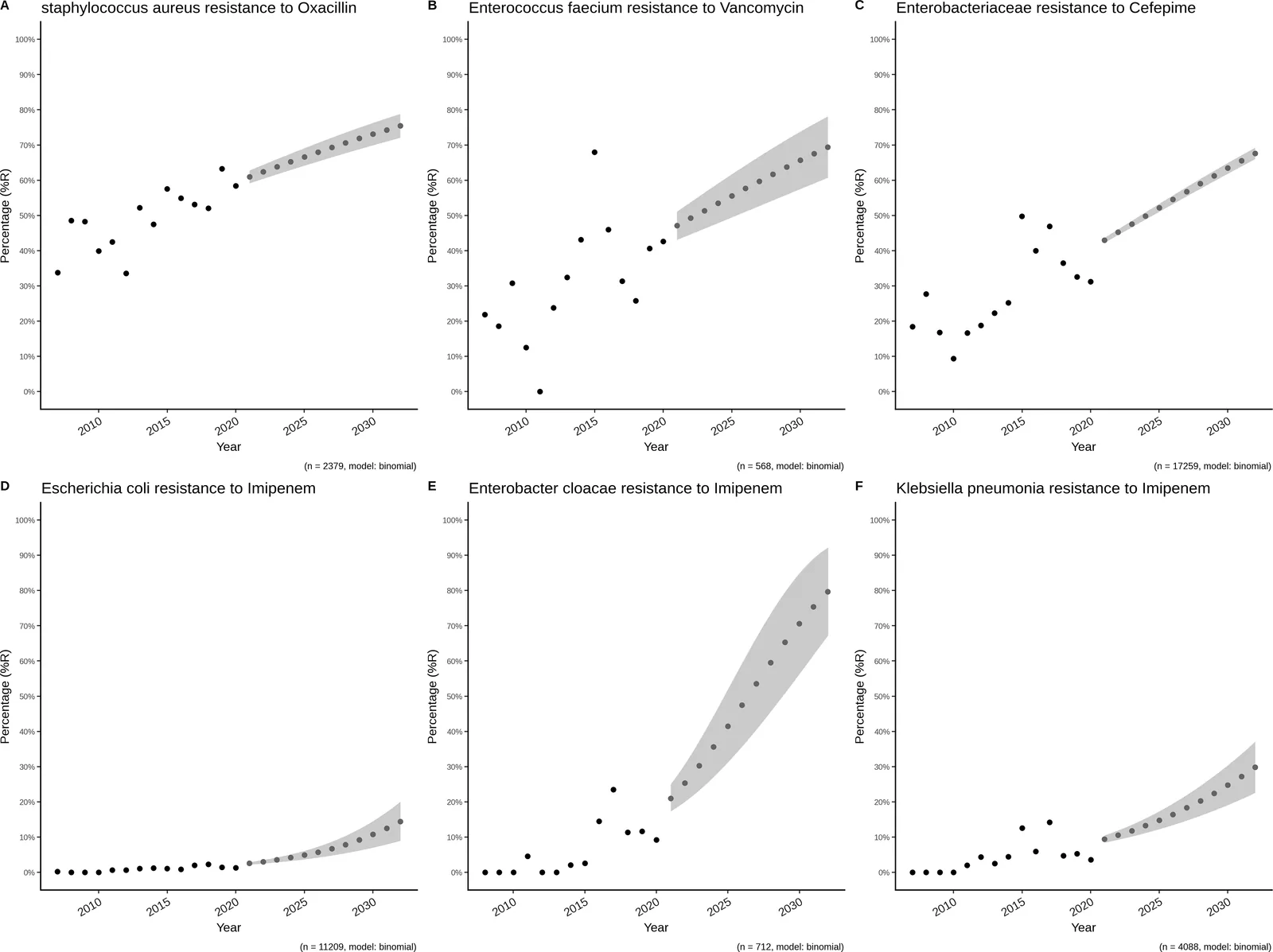
Observed and predicted antimicrobial resistance trends (2007–2032).

Binomial logistic regression models forecast alarming resistance escalations by 2030: MRSA is predicted to comprise ∼73% of *S. aureus* isolates (95% CI: 69.4–75.9%, *P* < .001), and VRE to represent 70% of *E. faecium* isolates (Figure 2). Among *Enterobacterales*, cefepime resistance is projected to reach 61.9% (95% CI: 60.2–63.5%, *P* < .001), with concerning carbapenem resistance increases anticipated in *Enterobacter* spp. (65%, 95% CI: 50.3–80.8%, *P* < .001) and *K. pneumoniae* (27.7%, 95% CI: 21.0–34.5%, *P* < .001). Conversely, carbapenem resistance in *A. baumannii-calcoaceticus* complex is predicted to decline (58.5%, 95% CI: 52–65.1%, *P* < .001), with no significant change in colistin resistance (*P* = .27).Conversely, carbapenem resistance in *A. baumannii-calcoaceticus* complex is predicted to decline by 2030 (estimate: 58.5%, 95% CI: 52–65.1%, *P* < .001), with no significant change projected in colistin resistance (*P* = .27).

## Discussion

This 16-year analysis reveals an alarming AMR trajectory at our tertiary cancer center, marked by a substantial burden of MDR and XDR pathogens and predictive models forecasting further deterioration by 2030. While GNB consistently predominated, the study identifies a dual-threat landscape: rising resistance among *Enterobacterales,* MRSA, and VRE, contrasted by a notable decline in resistant non-fermenting GNB (NFGNB), particularly *P. aeruginosa* and *A. baumannii-calcoaceticus* which serves as a critical finding. This decline temporally coincides with institutional changes around the start of the third period, including a major facility expansion (doubled bed capacity, quadrupled clinical space) and the formal activation of an antimicrobial stewardship committee.

In patients with resistant Gram-negative bacteria (GNB), chemotherapy-induced breakdown of mucosal barriers and frequent use of invasive devices are hypothesized to provide portals of entry for enteric organisms, a mechanism supported in the literature though not directly assessed in our data set.^12^ Resistant *Enterobacterales* is a growing concern. ESBL testing showed that more *E.coli* and *K. pneumonia* are getting resistance. This is coinciding with significantly increasing resistance to cephalosporins and carbapenems in the last period of the study (2018–2022). Carbapenem-resistant GNB phenotype is a major predictor of treatment failure and early mortality in cancer patients with systemic infections.^13^

The proportion of *S.aureus* and *E. faecium* resistant to oxacillin and vancomycin, respectively, are increasing, possibly reflecting changes within and outside the hospital. This increase is alarming, and it is predicted that in 10 years, almost three-quarters of *S.aureus* isolates will be resistant to oxacillin. One area of concern is the need to rely heavily on glycopeptides or oxazolidinone, as we currently have no approved alternatives. Luckily, we did not face vancomycin resistance among any of *S. aureus* isolates yet. It is not easy to correlate our findings with the literature. AMR varies over time and is related to the geographic location, study period, patient population studied, age-groups, use of prophylactic antibiotics and the accuracy in reporting antimicrobial resistance.^14^

Jordan has well-documented high rates of antibiotic resistance, local data highlight this burden: a study at a large public hospital reported high methicillin-resistant CoNS rates in hospitalized patients;^15^ 46% of community-acquired UTIs in children were caused by ESBL-producing bacteria;^16^ ESBL-producing E. coli was identified in 62% of UTI specimens across two tertiary hospitals;^17^ and 48% of environmental *E. coli* isolates from five tertiary hospitals were MDR, with 68% phenotypically identified as ESBL producers.^18^ MRSA prevalence is also notably high in Jordan, with a study across two tertiary hospitals finding that 73% of *S. aureus* clinical isolates harbored the mecA gene, confirming MRSA designation.^19^

Regarding multidrug-resistant (MDR) *Acinetobacter baumannii-calcoaceticus* complex isolates, a report from the largest military hospital in Jordan found that more than three-quarters of isolates were highly resistant to all antibiotics, including tigecycline, with only minocycline and colistin, the last-resort agents, retaining meaningful activity (resistance rates of 27% and 1.7%, respectively).^20^ A recent Jordanian study evaluated the resistance to newly introduced cephalosporines mainly ceftazidime-avibactam and ceftolozane-tazobactam, regrettably, found that some isolates had already developed resistance to these agents prior to their widespread use in the country.^21^

Rates of resistance seem to be higher in cancer wards. Praphash et al reported alarmingly high rates of resistance among adult cancer patients. In their study, half (49%) of all GNB were resistant to piperacellin/tazobactam and 28% were resistant to meropenem. Sensitivity to amikacin ranged from 24% to 60% and meropenem was reliable only in *E. coli* and *K. pneumoniae* (>90% sensitivity). Meropenem sensitivity was reported in only 66% and 39% of *pseudomonas* and *Acinetobacter* isolates, respectively. Interestingly, MRSA was reported only in 18% of their isolates.^22^

The SENTRY antimicrobial surveillance program, tracking resistance across hundreds of hospitals worldwide since 1997, reported a decline in MRSA prevalence in Western centers to approximately 39% (2013–2016) following a broader post2008 reduction in oxacillin resistance among *S. aureus*.^23^ This stands in stark contrast to our center, where MRSA prevalence continues to rise.^23^ This discrepancy suggests that regional factors, such as higher community colonization rates in Jordan or differences in decolonization practices, may be driving the institutional trend.

In contrast, the prevalence of MDR *Enterobacterales* increased from 6.2% in 1997 to 2000 to 15.8% in 2013 to 2016. MDR rates were highest among non-fermentative Gram-negative bacilli (GNB), and colistin was the only agent with predictable activity against *Acinetobacter baumannii-Acinetobacter calcoaceticus* complex (97% susceptible).^24^ ESBL rates among our patients were double those reported by SENTRY, which documented a peak MDR *Enterobacterales* prevalence of 15.8%, far below the 68.8% recorded in our study. Notably, SENTRY reported stable VRE prevalence post2012 (16.4% overall), which remains higher than our latest VRE rate of 13%.^24^

Patients with hematologic malignancies showed a more severe, unadjusted AMR profile than those with solid tumors, with leukemia and lymphoma patients exhibiting the highest XDR rates (5.6%–7.9%) versus 4.0% in carcinoma patients, an association, not evidence of an independent diagnosis effect. VRE was of particular concern, plausibly related to prolonged neutropenia, broad-spectrum antibiotic exposure, and central-line contact common in this population, though these variables were not captured or adjusted for in our data. The finding that one-quarter of blood enterococci were vancomycin-resistant nonetheless highlights a clinically relevant gap in current prophylactic and therapeutic strategies for hematologic patients.^12^

Urine cultures showed the highest density of Gram-negative bacteria, with a 63% MDR rate among GNB isolates; the high fluoroquinolone resistance (55%) in urine-derived *Enterobacterales* may relate to extensive ciprofloxacin use in prophylactic and outpatient settings, though this was not directly tested. Respiratory samples, in contrast, served as a primary reservoir for resistant NFGNB and MRSA, possibly linked to mechanical ventilation and upper airway colonization in chronically ill patients, consistent with prior reports,^25^ though this association was not directly assessed in our data.

Our 16-year, diagnosis- and site-stratified analysis, novel linkage to the cancer registry, and NHSN/CLSI-compliant methodology are real strengths, and the predictive modeling was a genuine attempt to move beyond a static antibiogram toward something more actionable for settings like ours without real-time EMR surveillance. Still, this study has several limitations, our 2030 projections are unvalidated extrapolations of past trends assuming those trends continue, and shifts in testing platforms over 16 years may have added some residual noise despite MIC harmonization. Ongoing efforts at our center aim to develop a real-time antibiogram dashboard integrated with cancer registry data to operationalize these findings in resource-limited settings.

## Conclusion

In conclusion, this study documents alarming and escalating resistance patterns across bacterial isolates at our institution, with significant variation by specimen site and cancer diagnosis. Empirical therapy for febrile neutropenia must be increasingly tailored to individual patient risk factors and site-specific antibiograms. Preserving the efficacy of last-resort agents such as colistin demands sustained institutional vigilance and a multifaceted response integrating rigorous clinical surveillance, evidence-based stewardship, and novel diagnostic technologies.

## Supporting information

10.1017/ash.2026.10888.sm001 Mubarak et al. supplementary material Mubarak et al. supplementary material

## Data Availability

The data sets generated and analyzed during the current study are available from the corresponding author upon reasonable request.

## Supplementary material

The supplementary material for this article can be found at https://doi.org/10.1017/ash.2026.10888.

## Table 1. Long description

The table presents data on the prevalence of different bacterial isolates over three study periods: 2007 to 2012, 2013 to 2017, and 2018 to 2022. The table has 10 rows and 4 columns. The columns are labeled ‘Bacterial isolates’, ‘2007 to 2012 N. (%)’, ‘2013 to 2017 N. (%)’, and ‘2018 to 2022 N. (%)’. The rows list different bacterial isolates and their respective counts and percentages for each period. Row 1: Escherichia coli, 3341 (33), 4125 (34), 5693 (33.5). Row 2: Klebsiella pneumoniae, 1163 (11.5), 1567 (12.9), 2345 (13.8). Row 3: Pseudomonas aeruginosa, 1048 (10.3), 1066 (8.8), 1559 (9.2). Row 4: Staphylococcus aureus, 762 (7.5), 837 (6.9), 1264 (7.4). Row 5: Acinetobacter baumannii-calcoaceticus, 706 (7.0), 529 (4.4), 685 (4.0). Row 6: Proteus mirabilis, 240 (2.4), 362 (3.0), 497 (2.9). Row 7: Enterococcus faecalis, 372 (3.7), 382 (3.1), 763 (4.5). Row 8: Viridans Group streptococci (VGS), 314 (3.1), 397 (3.3), 554 (3.3). Row 9: Coagulase-negative Staphylococcus (CONS), 2191 (21.6), 2876 (23.7), 3635 (21.4).

Navigate back to Table 1..

## Table 2. Long description

A table comparing antibiotic resistance profiles across three study periods: 2007-2012, 2013-2017, and 2018-2022. The table has 10 rows and 6 columns. Column headers are: Total Isolates, Gram-Negative, Percentage of total, MDR, XDR, and Gram-Positive. Row labels include the study periods and categories such as Total Isolates, Gram-Negative, Percentage of total, MDR, XDR, and Gram-Positive. Row 1: 2007-2012, Total Isolates, 13294; Gram-Negative, 8288; Percentage of total, 62.3 percent; MDR, 3402 (41 percent); XDR, 678 (8.1 percent); Gram-Positive, 5006. Row 2: 2013-2017, Total Isolates, 16021; Gram-Negative, 9869; Percentage of total, 61.6 percent; MDR, 3576 (36.2 percent); XDR, 478 (4.8 percent); Gram-Positive, 6152. Row 3: 2018-2022, Total Isolates, 22382; Gram-Negative, 13624; Percentage of total, 60.9 percent; MDR, 4936 (36.2 percent); XDR, 577 (4.2 percent); Gram-Positive, 8758. Row 4: 2007-2012, Gram-Positive, 5006; Percentage of total, 37.7 percent; MDR, 568 (11.3 percent). Row 5: 2013-2017, Gram-Positive, 6152; Percentage of total, 38.4 percent; MDR, 909 (14.7 percent). Row 6: 2018-2022, Gram-Positive, 8758; Percentage of total, 39.1 percent; MDR, 1474 (16.8 percent).

Navigate back to Table 2..

## Table 3. Long description

A table comparing antimicrobial resistance percentages across three periods: 2007-2012, 2013-2017, and 2018-2022. The table has 12 rows and 6 columns, including column headers and row labels. Column headers are: 2007-2012, 2013-2017, 2018-2022, and row labels include Staphylococcus aureus, Enterococcus species, Enterobacteriaceae, and NFGNB. Each row provides resistance percentages for specific antimicrobials. Notable trends include increasing resistance to oxacillin in Staphylococcus aureus, stable vancomycin resistance in Enterococcus species, and varying resistance levels in Enterobacteriaceae and NFGNB across different antimicrobials.

Navigate back to Table 3..

## Table 4. Long description

A table comparing antibiotic resistance profiles across different specimen sources. The table has 12 rows and 6 columns. The columns are labeled Diagnosis, Urine, Blood, Wound, Respiratory, and Others. The rows are labeled with different types of pathogens and resistance patterns. Row 1: Gram-Negative, 83.80 percent, 35 percent, 48 percent, 80.60 percent, 42.60 percent. Row 2: MDR, 63 percent, 53 percent, 46 percent, 35 percent, 49 percent. Row 3: XDR, 2.2 percent, 5.1 percent, 7.6 percent, 16 percent, 2.2 percent. Row 4: Gram-Positive, 16.20 percent, 65 percent, 52 percent, 19.40 percent, 57.40 percent. Row 5: MDR, 62 percent, 94 percent, 81 percent, 46 percent, 87 percent. Row 6: S. aureus, Oxacillin resistance, 52 percent, 55 percent, 58 percent, 60 percent, 56 percent. Row 7: Enterococcus species, Vancomycin resistance, 12 percent, 26 percent, 16 percent, NA, 21 percent. Row 8: Enterobacteriaceae, Amikacin, 3 percent, 7 percent, 5 percent, 6 percent, 24 percent. Row 9: Enterobacteriaceae, Ciprofloxacin, 55 percent, 53 percent, 49 percent, 42 percent, 46 percent. Row 10: Enterobacteriaceae, Piperacillin/tazobactam, 10 percent, 17 percent, 15 percent, 18 percent, 15 percent. Row 11: Enterobacteriaceae, Imipenem, 2 percent, 5 percent, 4 percent, 6 percent, 4 percent. Row 12: NFGNB, Amikacin, 26 percent, 35 percent, 30 percent, 58 percent, 35 percent. Row 13: NFGNB, Piperacillin/tazobactam, 27 percent, 37 percent, 33 percent, 62 percent, 34 percent. Row 14: NFGNB, Imipenem, 32 percent, 39 percent, 35 percent, 64 percent, 39 percent. Row 15: NFGNB, Colistin, 0 percent, 2 percent, 1 percent, 0 percent, 4 percent.

Navigate back to Table 4..

## Table 5. Long description

A table comparing resistance profiles by cancer diagnosis, focusing on percentages of tested isolates. The table has 10 rows and 7 columns. Column headers are Diagnosis, Carcinomas, CNS, Leukemia, Lymphomas, Bone and soft tissue. Row labels include Total, Gram-Negative, Percentage, MDR, XDR, Gram-Positive, Percentage, MDR, S. aureus, Oxacillin resistance, Enterococcus species, Vancomycin resistance, Enterobacteriaceae, Amikacin, Ciprofloxacin, Piperacillin/tazobactam, Imipenem, NFGNB, Amikacin, Piperacillin/tazobactam, Imipenem, Colistin. Each row provides specific data points for each diagnosis category. Notable trends include higher resistance percentages in leukemia patients for various antibiotics and higher XDR rates in hematologic malignancies and bone/soft tissue sarcomas.

Navigate back to Table 5..
