# Supplementary material for: Cumulative antibiogram at a tertiary cancer center in Jordan showing alarming antimicrobial resistance trends
Source: Antimicrob Steward Healthc Epidemiol. 2026 Oct 9;6(1):e311. doi: 10.1017/ash.2026.10888 (PMC13760307; doi:10.1017/ash.2026.10888)
Supplement: Supplementary file 1 [file S2732494X26108882sup001.docx]

Supplementary Table 1. Antibiogram of the 10 most common bacterial isolates over 3 periods (1: 2007-2012, 2: 2013:2017, 3: 2018:2022). Percentages shown represent sensitivity (susceptible+intermediate).

| Gram negative bacteria | Period | Ampicillin | Ampicillin/sulbactam | Piperacillin/tazobactam | Ceftriaxone | Ceftazidime | Cefepime | Ertapenem | Imipenem | Meropenem | Ciprofloxacin | Amikacin | Gentamicin | Aztreonam | Colistin | Tigecycline |
| --- | --- | --- | --- | --- | --- | --- | --- | --- | --- | --- | --- | --- | --- | --- | --- | --- |
| Acinetobacter calcoaceticus | 1 |  | 30% | 19% | 12% | 18% | 19% |  | 22% | 19% | 17% | 32% | 22% | 6% | 100% | 56% |
| Acinetobacter calcoaceticus | 2 |  | 30% | 25% | 14% | 22% | 25% |  | 26% | 26% | 22% | 34% | 33% |  | 100% | 69% |
| Acinetobacter calcoaceticus | 3 |  | 33% | 25% | 18% | 23% | 26% |  | 26% | 26% | 22% | 31% | 32% |  | 99% |  |
| Enterobacter | 1 | 2% | 22% | 90% |  |  | 91% | 98% | 99% | 99% | 86% | 97% | 86% | 76% |  | 97% |
| Enterobacter | 2 | 1% | 14% | 88% |  |  | 85% | 92% | 94% | 94% | 79% | 97% | 88% | 78% |  |  |
| Enterobacter | 3 |  | 1% | 81% |  |  | 78% | 86% | 91% | 92% | 71% | 94% | 90% | 71% |  |  |
| Escherichia coli | 1 | 12% | 54% | 94% | 56% | 58% | 84% | 100% | 100% | 100% | 44% | 99% | 67% | 59% |  | 100% |
| Escherichia coli | 2 | 8% | 56% | 78% | 48% | 49% | 66% | 99% | 99% | 99% | 40% | 99% | 71% | 49% |  | 99% |
| Escherichia coli | 3 | 10% | 56% | 89% | 46% | 46% | 64% | 96% | 98% | 98% | 36% | 98% | 77% | 48% | 90% |  |
| Klebsiella pneumoniae | 1 |  | 54% | 92% | 63% | 64% | 89% | 99% | 100% | 100% | 71% | 97% | 76% | 64% |  | 97% |
| Klebsiella pneumoniae | 2 |  | 52% | 86% | 54% | 54% | 69% | 93% | 95% | 95% | 65% | 95% | 74% | 55% |  | 92% |
| Klebsiella pneumoniae | 3 |  | 53% | 86% | 52% | 51% | 68% | 92% | 95% | 95% | 60% | 94% | 78% | 53% | 89% |  |
| Proteus mirabilis | 1 | 31% | 89% | 99% | 99% | 99% | 100% | 100% | 100% | 100% | 89% | 100% | 82% | 99% |  |  |
| Proteus mirabilis | 2 | 22% | 90% | 96% | 93% | 93% | 96% | 100% | 100% | 100% | 84% | 100% | 78% | 96% |  |  |
| Proteus mirabilis | 3 | 36% | 92% | 100% | 94% | 95% | 99% | 100% | 99% | 100% | 71% | 100% | 78% | 99% |  |  |
| Pseudomonas aeruginosa | 1 |  |  | 84% |  | 78% | 82% |  | 79% | 84% | 78% | 84% | 80% | 77% | 100% |  |
| Pseudomonas aeruginosa | 2 |  |  | 87% |  | 83% | 86% |  | 78% | 83% | 82% | 87% | 84% | 80% | 100% |  |
| Pseudomonas aeruginosa | 3 |  |  | 88% |  | 82% | 87% |  | 82% | 84% | 83% | 89% | 87% | 83% | 100% |  |

| Gram positive bacteria | Period | Ampicillin | Ampicillin/sulbactam | Piperacillin/tazobactam | Ceftriaxone | Minocycline | Tigecycline | Benzylpenicillin | Oxacillin | Erythromycin | Clindamycin | Vancomycin | Teicoplanin | Linezolid |
| --- | --- | --- | --- | --- | --- | --- | --- | --- | --- | --- | --- | --- | --- | --- |
| CONS | 1 |  |  |  | 25% | 91% | 100% | 5% | 24% | 22% | 51% | 100% | 100% | 100% |
| CONS | 2 |  |  |  | 27% | 100% | 98% | 2% | 25% | 22% | 56% | 100% | 100% | 100% |
| CONS | 3 |  |  |  |  | 98% |  | 4% | 19% | 21% | 54% | 100% | 99% | 99% |
| Enterococcus spp. | 1 | 70% | 100% | 70% |  | 64% | 100% | 66% |  |  |  | 95% | 97% | 100% |
| Enterococcus spp. | 2 | 63% | 100% | 63% |  | 33% |  | 60% |  |  |  | 87% | 92% | 100% |
| Enterococcus spp. | 3 | 68% | 100% | 68% |  | 46% |  | 64% |  |  |  | 87% | 87% | 98% |
| Staphylococcus aureus | 1 |  |  |  | 63% | 96% | 100% | 4% | 63% | 65% | 71% | 100% | 100% | 100% |
| Staphylococcus aureus | 2 |  |  |  | 57% | 99% | 99% | 2% | 50% | 64% | 72% | 100% | 100% | 100% |
| Staphylococcus aureus | 3 |  |  |  |  | 99% |  | 2% | 43% | 65% | 70% | 100% | 100% | 100% |
| Viridans Group Streptococcus (VGS) | 1 | 100% |  | 100% | 86% |  |  | 88% |  | 48% | 78% | 100% |  | 100% |
| Viridans Group Streptococcus (VGS) | 2 | 100% |  | 100% | 84% |  |  | 91% |  | 45% | 71% | 100% |  | 100% |
| Viridans Group Streptococcus (VGS) | 3 | 100% |  | 100% | 87% |  |  | 91% |  | 46% | 68% | 99% |  | 100% |
